# Reconstruction of Gap-Free Land Surface Temperature at a 100 m Spatial Resolution from Multidimensional Data: A Case in Wuhan, China

**DOI:** 10.3390/s23020913

**Published:** 2023-01-12

**Authors:** Zefeng Wu, Hongfen Teng, Haoxiang Chen, Lingyu Han, Liangliang Chen

**Affiliations:** 1 School of Environmental Ecology and Biological Engineering, Wuhan Institute of Technology, Wuhan 430205, China; 2 Key Laboratory of Agricultural Remote Sensing and Information System, Hangzhou 310058, China; 3 College of Environmental and Resource Sciences, Zhejiang University, Hangzhou 310058, China

**Keywords:** land surface temperatures, random forest regression, reconstruction, Landsat 8, spatial feature

## Abstract

Land surface temperatures (LST) are vital parameters in land surface–atmosphere interactions. Constrained by technology and atmospheric interferences, LST retrievals from various satellite sensors usually return missing data, thus negatively impacting analyses. Reconstructing missing data is important for acquiring gap-free datasets. However, the current reconstruction methods are limited for maintaining spatial details and high accuracies. We developed a new gap-free algorithm termed the spatial feature-considered random forest regression (SFRFR) model; it builds stable nonlinear relationships to connect the LST with related parameters, including terrain elements, land coverage types, spectral indexes, surface reflectance data, and the spatial feature of the LST, to reconstruct the missing LST data. The SFRFR model reconstructed gap-free LST data retrieved from the Landsat 8 satellite on 27 July 2017 in Wuhan. The results show that the SFRFR model exhibits the best performance according to the various evaluation metrics among the SFRFR, random forest regression and spline interpolation, with a coefficient of determination (R^2^) reaching 0.96, root-mean-square error (RMSE) of 0.55, and mean absolute error (MAE) of 0.55. Then, we reconstructed gap-free LST data gathered in Wuhan from 2016 to 2021 to analyze urban thermal environment changes and found that 2020 presented the coolest temperatures. The SFRFR model still displayed satisfactory results, with an average R^2^ of 0.91 and an MAE of 0.63. We further discuss and discover the factors affecting the visual performance of SFRFR and identify the research priority to circumvent these disadvantages. Overall, this study provides a simple, practical method for acquiring gap-free LST data to help us better understand the spatiotemporal LST variation process.

## 1. Introduction

To understand the impacts of human activities in highly spatially heterogeneous areas and to understand the response to global environmental changes, the land surface temperature (LST) has been widely used for various applications [1], including in drought-monitoring research [2], evapotranspiration estimations [3], urban thermal environment research [4], and global climate change research [5].

The development of remote sensing technologies has greatly facilitated the acquisition of LST data [6]. The currently available satellite images from which LST can be retrieved include the images of the Landsat series, Moderate Resolution Imaging Spectroradiometer (MODIS) sensor data, and Sentinel satellites. Various algorithms have been proposed for retrieving LST data from satellite images according to different sensors [7], including the single thermal infrared (TIR) channel method [8,9], multichannel methods [10], and split-window algorithms [11,12]. LST retrievals are characterized by their wide spatial coverage, long-term observation periods, and diverse spatial resolutions. For example, the Landsat series of satellites provide data at a high spatial resolution reaching 30 m with a revisit period of 16 days, while MODIS-series products offer daily LST data with relatively low spatial resolutions of 1 km. However, due to technical and air condition constraints, retrieving LST data from a single sensor always returns unreal values; for example, clouds will absorb the thermal infrared emissions and leave an incorrect LST. This leads to negative effects when using these data in LST research [13]. Hence, it is difficult to collect cloud-free satellite images from which to obtain the LST in a given study area and within a limited period.

To date, several algorithms have been developed for acquiring gap-free LST data. These methods can be roughly divided into three categories [13,14,15]. The first category includes spatial information-based methods. Because of the spatial continuity and autocorrelation traits possessed by the LST, these methods are commonly used, especially in the research field of gap-filling data retrieved from Landsat 7 satellites [16,17], to reconstruct target pixels using the information of adjacent cloud-free pixels, such as through spline curve interpolations [18,19], geostatistical interpolation methods [20], and inverse distance weighting [21]. However, the performance of these spatial information-based methods is weakened due to their reliance on only spatial information while neglecting temporal information when the missing data extend over a relatively large range [22,23]. The second category comprises multitemporal information-based methods, which can be used to reconstruct gap-free pixels representing the same region at different times. Xu et al. [24] developed the harmonic analysis of the time series algorithm to reconstruct eight-day MODIS LST data based solely on time series LST data retrieved from Landsat satellite images. Although multitemporal information-based methods work well in regions with large quantities of missing data, the performance of these methods is weakened when large gaps exist in the acquisition time of multitemporal data due to land cover type variations [23,25]. The last category refers to hybrid methods derived based on spatiotemporal information; these methods are also regarded as the most promising methods [14] because they incorporate abundant information. Data fusion is a classical method belonging to this category. Proposed by Feng et al. [26], the spatial and temporal adaptive reflectance fusion model algorithm has been widely popular for generating high spatiotemporal resolution LST by integrating Landsat and MODIS LST data [27,28]. The method uses all the data at time t1 and coarse resolution data at time t2 to predict the fine resolution data at time t2. To decrease the systematic error caused by the heterogeneous surface, three weight functions, including spectrum, time, and space, are introduced. However, it is sensitive in heterogeneous areas, and the accuracy of its predictions remains to be fully assessed [29]. Traditional methods are widely used to obtain gap-free LST data, but the performances of these methods are affected by multiple factors, such as limited effective original data, land cover changes, sensitivity to high spatial heterogeneity, and applied accuracy verification [15]. Recently, machine learning models have been used to better describe nonlinear relationships due to the complicated formation of LST data [30]. Zhao et al. [31] applied a random forest (RF) regression model to reconstruct gap-free LST data in 2015 in southwestern Europe using Terra/MODIS daytime observations by building nonlinear links between the LST and related parameters. Their method achieved a correlation coefficient (R^2^) of 0.94 with a good visual performance. Xiao et al. [32] constructed an RF regression model to reconstruct a time series gap-free LST in Chongqing City, and the results showed that R^2^ value between the reconstructed LST and in situ LST measurements reached 0.89. Although machine learning models have good effects with respect to evaluation metrics and visual performance, their results are limited by the quality of the training dataset. Moreover, it is also hard to find appropriate auxiliary spatial parameters with fine resolution given the complex formation of the dependent variables.

Hence, in response to these challenges in acquiring high-resolution gap-free LST, we propose an algorithm that improves upon the RF regression model, which is termed the spatial feature-considered random forest regression (SFRFR) model. The characteristics of RF regression and Kriging regression are combined to construct this method. Specifically, RF regression is first used to improve the quality of the original data and obtain the spatial LST feature; then, a stable nonlinear link is built and connected to the LST with several spatiotemporal parameters that are directly related to the LST, including terrain [33], population density (PD) [34], land use and land cover change (LUCC) [35], soil texture (in terms of the clay, silt, and sand contents, ST) [36], enhanced vegetation index (EVI) [37], solar zenith angle (SZA) [38], and middle-infrared surface reflectance (MIR) [13] data as well as the LST spatial feature of the LST. Finally, Kriging regression is applied to further narrow the residuals. We reconstructed gap-free LST data in Wuhan obtained on 26 July 2017 and tested the model performance. Then, we used the method to reconstruct the gap-free summertime LST in Wuhan from 2016 to 2021 and analyzed the temporal and spatial variations in the urban thermal emissions. In addition, we reconstructed the LST at a lower resolution of 250 m. Comparing the results at the 100m resolution, it could be found that the SFRFR method still demonstrated the best performance, with a more noticeable improvement at the lower resolution. Through a series of repeated modeling experiments, the SFRFR model still possesses the best performance among the assessed methods.

## 2. Materials and Methods

### 2.1. Study Area

**Figure 1:**
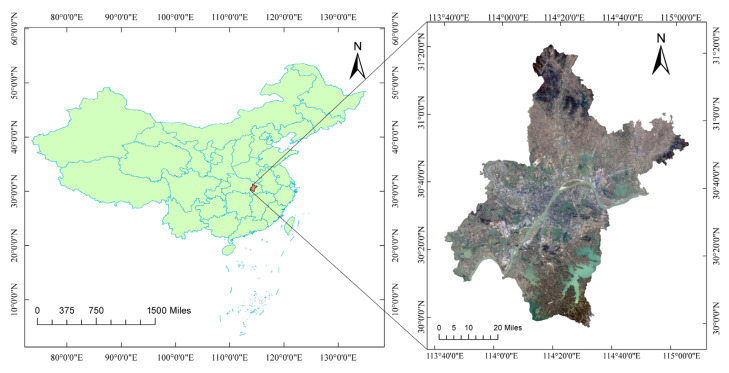
Location of the study area in China; standard false-color satellite image of Wuhan, composed of bands 4, 3, and 2 from the Landsat 8 satellite.

Wuhan, located in central China and covering the geographical range of 29°58′–31°22′ N, 113°41′–115°05′ E, was selected as the study area (Figure 1). The maximum distance from the east to the western edges of the study area is 134 km, while the maximum distance from the north to south edges is 155 km. The study area belongs to the subtropical monsoon climate zone and is known as the “Furnace City” due to its abundant rainfall, four distinct seasons, relatively hot summers, and other characteristics.

### 2.2. Data Acquisition and Processing

The Landsat program was launched by the United States Geological Survey and National Aeronautics and Space Administration. It aims to acquire satellite imageries of Earth and perform related research [39]. Carrying the Operational Land Imager (OLI), the Thermal Infrared sensor (TIRs), and Enhanced Thematic Mapper Plus (ETM+) instruments, Landsat-series satellites provide images at a 30 m spatial resolution and 16 day temporal resolution. In this study, all LST data were obtained through the practical single-channel (PSC) algorithm, in which the RMSE reached 1.1 K according to the validation results [40]. These PSC LST products can be obtained on the web at https://psc-app.com (accessed on 3 April 2022). Because the PSC algorithm uses a TIRs band, which has a 100 m spatial resolution, to retravel the LST, all LST data were exported and resampled at 100 m resolution for the convenience of calculation. The Landsat 8 LST data collected on 26 July 2017 were applied to reconstruct the missing data in this study.

MODIS is the sensor mounted on the Terra and Aqua satellites; it captures data in 36 spectral bands, with spatial resolutions ranging from 250 m to 1 km and temporal resolutions of 1–2 days [41,42]. MODIS data can be collected from the Earth Explorer website (https://earthexplorer.usgs.gov/, accessed on 3 April 2022). In this study, the MOD13Q1 product, which provides data generated every 16 days at a 250 m spatial resolution, is chosen to obtain parameters related to the LST, including the EVI, SZA, and surface reflectance of the MIR band (Table 1) [43]. The EVI is calculated similarly to the normalized difference vegetation index (NDVI) but uses additional wavelengths of light to correct for the inaccuracies of the NDVI [37]. Therefore, we selected the EVI to make connections with the LST. Soil moisture is an important surface parameter that influences LST variation by affecting transpiration related to LST [44]. The MIR band, which proves the positive linear relationship between soil moisture, was selected as a proxy to indicate the change in soil moisture [13]. Peng et al. [38] used the SZA as a parameter to downscale LST. The SZA refers to the angle between the incident light direction and the zenith direction. When the altitude angle of the sun is 90°, it means that the sun is at the zenith and the SZA is 0. In this case, the amount of thermal radiation that is transmitted to the surface ground is the largest.

The digital elevation model (DEM), slope, LUCC, latitude, and longitude were also selected (Table 1). Among them, the DEM, which relates to the LST due to terrain fluctuations having an effect on solar radiation absorption [33,45], was collected from the Advanced Land Observing Satellite (ALOS) at a 12.5 m spatial resolution, and the data from this satellite can be acquired on the following website: https://search.asf.alaska.edu (assessed on 27 February 2022). Then, the 12.5 m slope data, which determines the receipt of surface radiation [31], were obtained based on the DEM data. Because the spatial distribution of LST is affected when the land coverages differ [46], the 30 m LUCC data freely obtained on the internet (https://zenodo.org/record/4417810, assessed on 4 April 2022) were selected to promote the robustness of the SFRFR results. Latitude and longitude, which represent differences in solar radiation and the impact of moisture conditions on the LST [47], were obtained through the function Add XY coordinates in ArcGIS 10.8 software.

The ST data include the soil’s clay, silt, and sand contents, which have been reported to be related to the LST and were thus chosen as explanatory variables (Table 1). Sayão et al. [36] found that there was an obvious trend between soil textures and LST; the contribution of soil textures had a similar pattern to the elevation and reflectance of thermal infrared bands. Our study area is abundant with water resources; to decrease the errors, we assigned zero value to water bodies in the soil texture. The data are freely available on the web (https://soilgrids.org/, assessed on 18 March 2022) at a spatial resolution of 250 m. The 100 m PD data which was positive related to the LST representing the study area were acquired from the web (https://worldpop.org/, assessed on 28 March 2022); this website offers incomplete data representing China.

### 2.3. Methodology

#### 2.3.1. Spline Interpolation Method

The Spline interpolation (SI) method model is a classical method based on spatial information. It can be regarded as applying a mathematical function to fit a surface with minimal curvature through known points. Serkan et al. [48] used the SI method to estimate the LST in the regions polluted by clouds and then combined deep learning methods to generate MODIS gap-free LST data. This method is used widely due to its simplicity and convenience; in this way, missing data can be reconstructed using only the inherent spatial information. The method is suitable for generating gently varying surfaces. The SI method has two classifications: regularized and tension classifications. In this study, the regularized type was chosen to fill the gaps. The method creates a smooth, gradually changing surface with values that may lie outside the range of the sample data [49].

#### 2.3.2. RF Regression Model

The RF regression model, an ensemble algorithm model constructed by taking multiple unrelated decision trees (DTs) as the basic unit, has been widely used to solve regression and classification problems in the remote sensing field [50]. In the regression model, the bootstrap method is first used to randomly sample data with replacement to form a DT. After each tree has been trained and predicted, the results of the RF regression model are obtained by averaging the predictions of all DTs. These DTs consist of nodes and edges. On each split, a sample feature with the most abundant information is chosen as a new internal node, and the selection of sample features is dependent on different rules, including the iterative dichotomiser-3 (ID3) method [51] and classification and regression tree (CART) method [52]. In each random sampling modeling, though the features participating in the modeling are also random (the number of features depends on the setting of the algorithm parameters), multiple repeated sampling progress can guarantee that each feature is not absent from the modeling. The nonlinear relationship between features and the dependent variable is further constructed. The RF method has many advantages. First, its complete random sampling characteristics make the resulting predictions more reliable. Second, the DTs can adapt to the splitting rule to flexibly construct nonlinear relationships between the predictor and parameters. Moreover, each independent tree performs these calculations in parallel, thus saving computing time. In this study, the bootstrap aggregation strategy sampled 70% of the data in each iteration as the training dataset, while the remaining 30% of the data were applied as the out-of-bag (OOB) data for validation.

#### 2.3.3. The Spatial Feature-Considered Random Forest Regression Model

**Figure 2:**
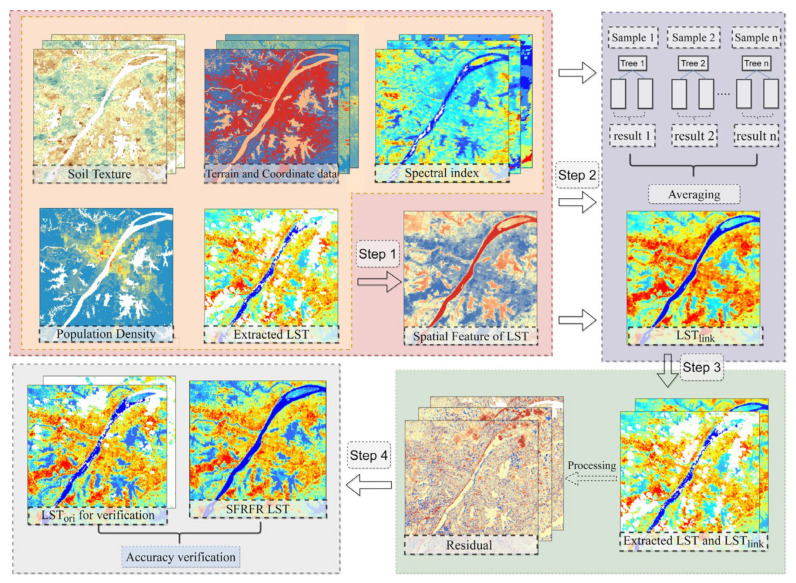
Flowchart of the proposed method for reconstructing missing data.

In this study, we considered the spatial feature of LST as a parameter to participate in the modeling. Then, we corrected the predicted LST using the residual between the original and predicted LST to obtain the ultimate LST value. A summary of the gap-free LST reconstruction process schematic is shown in Figure 2. The details can be divided into the following steps.

1. Step 1: Obtain the spatial features of the original LST

The LST is more related to the surrounding pixels than to distant pixels due to its spatial continuity and autocorrelation characteristics [48]. Hence, the spatial feature of the LST can be defined as the weighted LST value determined by the distance between the center pixel and adjacent pixels. The higher the assigned weight is, the closer the spatial distances of the surrounding pixels to the central pixel. The spatial feature of the LST can be expressed as follows:

(1) $$SFLST_{m}=\frac{\sum_{n=1}^{N}\frac{1}{d_{n}^{2}}LST_{n}}{\sum_{n=1}^{N}\frac{1}{d_{n}^{2}}}$$

where $SFLST_{m}$ denotes the spatial feature of the LST at the central pixel *m*, N represents the total pixels while n represents the adjacent pixel n near the central pixel m, $d_{n}^{2}$ represents the spatial distance between pixels m and n, and $LST_{n}$ is the LST value at pixel n.

Additionally, as the existence of missing data is caused by complex factors such as rain and clouds, it is impossible to obtain the spatial feature of the LST in null areas. The RF regression model was first used to enhance the quality of the original LST by reconstructing these missing parts.

2. Step 2: Construction of the stable nonlinear link

In this step, the RF regression model is improved by incorporating the spatial feature of the LST calculated in Step 1 into the modeling process; then, the predicted LST generated by the link can be presented as follows:

(2) $$LST_{link}=f\left(x_{terrain},x_{PD},x_{LUCC},x_{mir},x_{index},x_{ST},SFLST\right)$$

where $LST_{link}$ is the LST built by the link, $f\left(\ldots \right)$ denotes the nonlinear function, $x_{terrain}$ includes the DEM, slope, and coordinates, $x_{PD}$ is the PD data, $x_{LUCC}$ is the land use and land cover change data, $x_{mir}$ denotes the surface reflectance of the MIR band, $x_{index}$ is the collection of SZA and EVI data, $x_{ST}$ includes the soil clay, silt, and sand contents, and *SFLST* is the spatial feature of the LST.

3. Step 3: Processing of residuals

Based on the complex LST formation and limited predictive competence of the model, residuals between the original and predicted LST always exist. The smaller this residual is, the better the model performance. Residuals are widely applied to correct the predicted LST and narrow the gap between the predicted and original LST [53,54]. The residual can be expressed as follows:

(3) $$\varepsilon_{1}=LST_{ori}-LST_{link}$$

where $\varepsilon_{1}$ denotes the residual between the original and predicted LST and $LST_{ori}$ is the original LST value.

As the original LST data contain missing pixels, the $\varepsilon_{1}$ values also correspondingly contain default data. Here, a Kriging regression interpolation is applied to reconstruct the missing data:

(4) $$\varepsilon_{2}=\overset{n}{\underset{i=1}{\sum }}\lambda_{i}\varepsilon_{i}$$

where $\varepsilon_{2}$ is the prediction of Kriging interpolation, $\lambda_{i}$ represents the weight at pixel i, $\varepsilon_{i}$ denotes the observed value at pixel i, and n denotes the total-pixel value.

Then, coupling the two residuals, the gap-free residual $\varepsilon_{3}$ is obtained.

4. Step 4: Acquire the ultimate gap-free LST

Following the $\varepsilon_{3}$ correction, the predictions constructed by the SFRFR model were further improved. The corrected LST, which were termed as the SFRFR LST, were more similar to the original LST. These LST can be described as follows:

(5) $$LST_{sfrfr}=LST_{link}+\varepsilon_{3}$$

where $LST_{sfrfr}$ is the SFRFR-predicted LST, $LST_{link}$ denotes that the predicted LST comes from Step 2, and $\varepsilon_{3}$ represents the gap-free residual obtained from Step 3.

### 2.4. Cloud Mask for Verification

**Figure 3:**
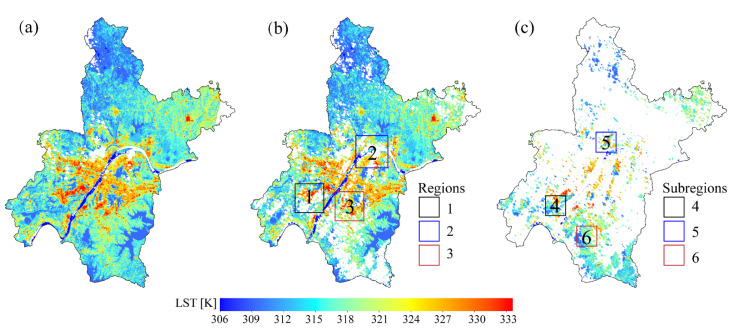
The 100 m LST data retrieved from Landsat 8 images by the PSC platform: (**a**) original LST; (**b**) extracted LST; and (**c**) masked LST. Regions 1, 2, and 3 and subregions 4, 5, and 6 show the specific areas selected for comparison.

Due to limited ground-station measurement coverage, the validation accuracy is weakened when the missing-data region is located far away from a station. The accuracy of these gap-filled missing areas is often assessed in reference to the evaluation metrics of other unpolluted regions because of the lack of an original LST in these regions. Therefore, to explore the generalization ability of the models more precisely, as shown in Figure 3, a random data mask was applied to extract the original LST. We termed these LST values in the mask regions as masked LSTs, and it has no contribution to the modeling process; once the predicted LST have been generated, the masked LST were used to verify the results. The remaining original LST, named the extracted LST, participated in the modeling process. It should be noted that after extracting some of the original LST and dividing the corresponding datasets, only half of the original LST were actually involved in the modeling.

To explore the performances of the gap-free LST intuitively reconstructed by the three methods, we selected three regions (regions 1, 2, and 3 in Figure 3b) in which extracted LST pixels and missing data remain. Then, we magnified the details, and three other subregions (subregions 4, 5, and 6 in Figure 3c) were chosen to analyze the spatial texture differences and explore the generalization ability of the predictions.

### 2.5. The Further Application and Exploration of SFRFR

#### 2.5.1. An Application for Analyzing Urban Thermal Environment Changes

To better assess the stability and practicability of the method and observe the urban thermal environment variations in the study area, we reconstructed gap-free summertime LST data for Wuhan from 2016 to 2021 using the SFRFR model. Due to the existence of LST fluctuations caused by extreme climate conditions, even when considering the Landsat 7-LST data and improving the time resolution to 8 days, the number of images with less cloud pollution was still deficient. Most images severely affected by clouds returned LST data with partially incorrect values, which needed to be removed. Therefore, we selected three satellite images with relatively less cloud coverage each year to reconstruct the gap-free LST. The LST retrieved from these images were averaged to obtain the summertime LST of the current year. Table 2 shows the original information of the selected images. Data gaps due to cloud cover and scan line corrector (SLC) failure can be regarded as loss mechanisms of valid data, so it is feasible to use the SFRFR model to reconstruct gap-free LST data from Landsat 7 images.

#### 2.5.2. Further Exploration of Model Performance

The random forest model is a classical machine learning algorithm. The predicted performance is positively correlated with the training sample size to some extent. At the spatial resolution of 100 m, a large quantity of data reaches more than 1 million involved in the model. In this case, the RF model may overfit and the generalization ability may decrease, which means that the model performance will be good but with less potential to improve. Reducing the training samples is a practical method for preventing overfitting, but simply reducing the number of training datasets may lead to some important details being absent from the model. To better explore the SFRFR performance and the improvement led by spatial features, we changed the spatial resolution of Landsat-LST data to 250 m through bilinear interpolation in Arcgis 10.8 software, and the dataset used for modeling was correspondingly reshaped to only one-tenth of the original. The evaluation metrics between the original LST against the predictions and visual performance were assessed.

### 2.6. Validation Method

To examine the robustness of the proposed model, three classical evaluation indexes, the R^2^, RMSE, and MAE, were adopted to verify the model performances. The indexes can be expressed as follows:

(6) $$R^{2}=1-\frac{\sum {\left(LST_{o}-LST_{P}\right)}^{2}}{\sum {\left(\bar{LST_{o}}-LST_{P}\right)}^{2}}$$

(7) $$RMSE=\sqrt{\frac{1}{n}\overset{n}{\underset{i=1}{\sum }}{\left(LST_{o}-LST_{p}\right)}^{2}}$$

(8) $$MAE=\frac{1}{n}\overset{n}{\underset{i=1}{\sum }}\left|\left(LST_{o}-LST_{P}\right)\right|$$

where $LST_{o}$ represents the original LST, $LST_{P}$ denotes the predicted LST, n is the total count of pixels, i denotes the $i_{-th}$ pixel in the evolution, and $\bar{LST_{o}}$ is the average value of the original LST.

Furthermore, because the proposed method and RF regression are both machine learning algorithms, the importance score ranking, a reliable proxy, was applied to measure the modeling contributions of the spatiotemporal parameters. To better verify and compare the performance of the proposed method, two traditional data-filling methods, the SI and RF regression models, were selected in this paper.

In the validation process of the time series reconstructed gap-free LST, ground-station data were used to verify the accuracy. We selected five ground stations, including Caidian, Huangpi, Jiangxia, Wuhan, and Xinzhou station, to validate the LST variation trend. For the machine learning model, a portion of the data called the out-of-bag data (OOB data) was left out to perform accuracy verification when modeling, and the verification scores, also called the OOB scores [53], were used to evaluate the accuracy of the results. We selected the R^2^ and the mean square error (MSE) as the evaluation metrics to assess the applicability and stability of the SFRFR model.

## 3. Results

### 3.1. Results of the Evaluation Metrics

**Figure 4:**
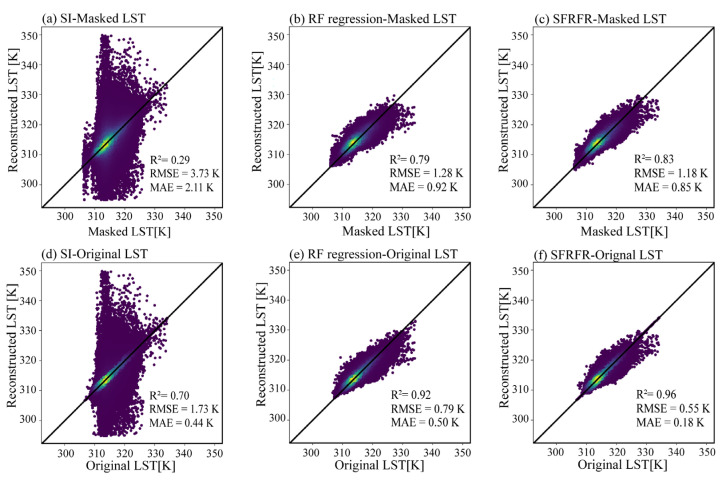
Scatter density plots between the LST predicted by the three methods and the observed LST: (**a**–**c**) LST plots constructed by the SI, RF regression, and SFRFR models against the masked LST; (**d**–**f**) LST plots constructed by the SI, RF regression, and SFRFR models against the original LST.

The scatter density plots of the LST predicted by the SFRFR, RF regression, and SI methods against the masked LST are shown in Figure 4a–c, while Figure 4d–f shows plots of the predicted LST against the original LST. Overall, the predicted performances of the SFRFR and RF regression methods are in good and similar condition, as is reflected by most scatter points being clustered along the 1:1 line, while the scatter points obtained against the SI are more randomly distributed. It is not difficult to see that the SFRFR results display more scatters distributed around the 1:1 line, indicating that the LST predicted by SFRFR have lower extremes than the RF regression results. In the masked LST region, the SFRFR results have the best metric values of R^2^ (0.83), RMSE (1.18), and MAE (0.85), followed by the RF regression results (R^2^, RMSE, and MAE values of 0.79, 1.28, and 0.92, respectively) and SI results (R^2^, RMSE, and MAE values of 0.29, 3.73, and 2.11, respectively). By comparing the evaluation metrics between the masked LST and original LST regions, the performances among the three models can be seen to have improved to various degrees. The SI method exhibited the best improvement among the three models, although it was still tertiary to the SFRFR and RF regression, and a certain gap still remained regardless of the dataset used for the validation. Overall, reaching values of 0.96 for R^2^, 0.55 for RMSE, and 0.18 for MAE, the SFRFR model is still the prime method among the three assessed methods.

### 3.2. Performance of the Reconstructed Gap-Free LST

**Figure 5:**
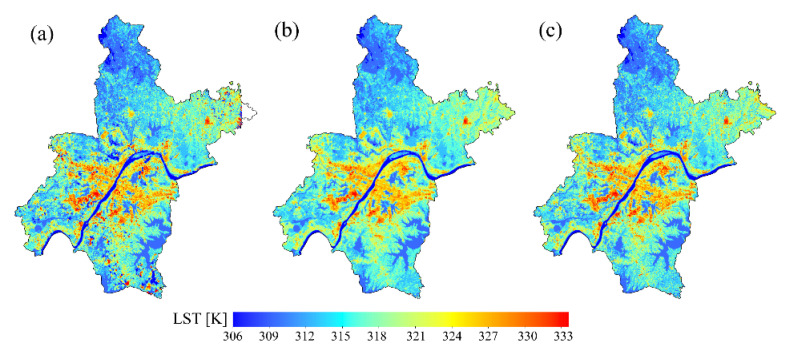
Performances of the predictions and the original LST: (**a**) SI LST, (**b**) RF regression LST, and (**c**) SFRFR LST.

The visual performances of the LST predictions are shown in Figure 5. The three methods seem to have good visual performances and simulate the LST with spatial distribution textures similar to the surrounding pixels. The SFRFR and RF regression methods can completely fill missing data, whereas the northeast corner of the study area still lacks data in the SI prediction results (Figure 5a). High temperatures are concentrated in urban areas, with the highest LST reaching 334.3 K, while low temperatures are located in areas with natural coverage, with the lowest LST being 303.55 K. Moderate LST values are observed at the junctions of urban areas and other coverage types. The spatial LST variations are consistent with the LUCC variations, suggesting that all three models work well when simulating LST data.

**Figure 6:**
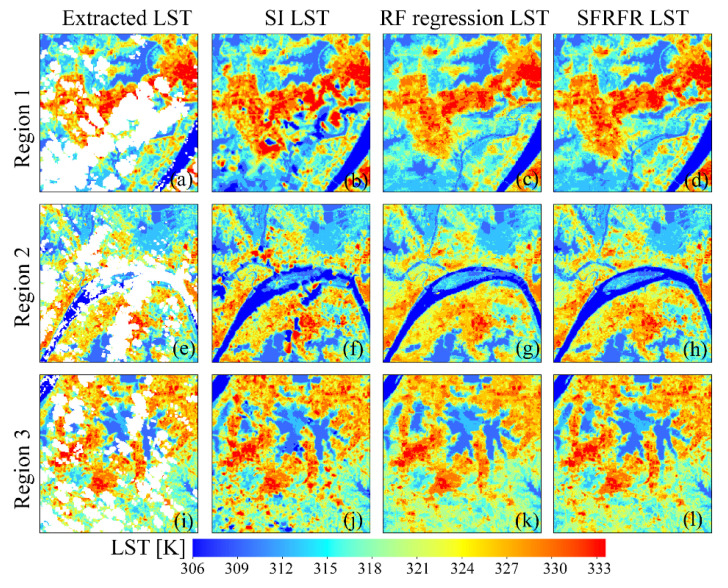
Visual performances of the reconstructed LST generated by the three models and the extracted LST in regions 1, 2, and 3: (**a**) extracted LST in region 1, (**b**) SI LST in region 1, (**c**) RF regression LST in region 1, (**d**) SFRFR LST in region 1, (**e**) extracted LST in region 2, (**f**) SI LST in region 2, (**g**) RF regression LST in region 2, (**h**) SFRFR LST in region 2, (**i**) extracted LST in region 3, (**j**) SI- LST in region 3, (**k**) RF regression LST in region 3, and (**l**) SFRFR LST in region 3.

Figure 6 presents the ability of the models to reconstruct missing areas. Although both the SFRFR and RF regression methods produce satisfactory visual results with respect to the gap-filling missing pixels, we found that the former method is superior to the latter, as illustrated in Figure 6f,g; the figure shows that the SFRFR model performs better than the RF regression model in the local area of the Changjiang River. The spatial variations in the reconstructed gap-free LST predicted by these two models against the surrounding extracted LST are stable, without sudden LST decreases or increases. In contrast, the SI method has a certain effect on reconstructing the gap-free LST when the missing data extend in a slight range; only in this situation is the spatial variation in the reconstructed LST against the surrounding original LST stable. However, when the missing data extend across a relatively large range, extreme outliers can be visually found, representing a consistent phenomenon in which the SI method tends to predict more extreme values in error.

**Figure 7:**
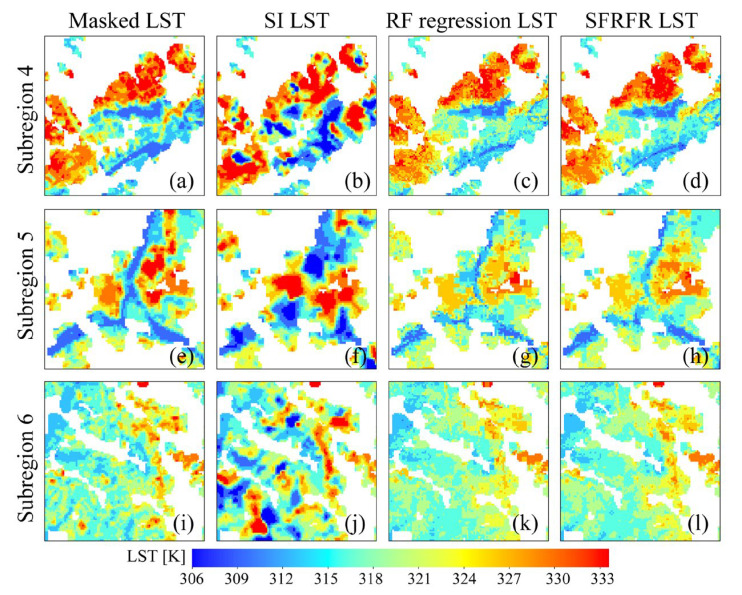
Detailed performances of the predicted LST in the subregions of the masked LST: (**a**) masked LST in subregion 4, (**b**) SI LST in subregion 4, (**c**) RF regression LST in subregion 4, (**d**) SFRFR LST in subregion 4, (**e**) masked LST in subregion 5, (**f**) SI LST in subregion 5, (**g**) RF regression LST in subregion 5, (**h**) SFRFR LST in subregion 5, (**i**) masked LST in subregion 6, (**j**) SI LST in subregion 6, (**k**) RF regression LST in subregion 6, and (**l**) SFRFR LST in subregion 6.

Figure 7 shows the detailed difference in the simulated LST built by the models. The continuity of the spatial LST distribution is still significant in the RF regression and SFRFR results, indicating that the spatial variations in the surface thermal properties are accurately captured by the models. The SI-predicted LST data tend to present more extreme values and great spatial fluctuations. The two machine-learning-based methods present similar spatial texture features, as shown in Figure 7k,i; in the figure, it can be seen that the visual performance of the SFRFR model is closer to the masked LST than the RF regression results.

### 3.3. The Residuals and Importance Scores Ranking

**Figure 8:**
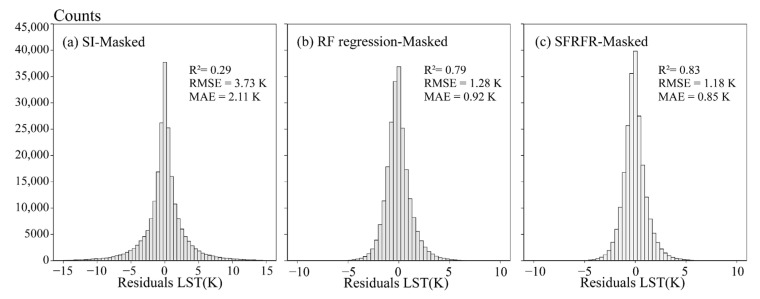
Distributions of residuals between the estimated and masked LST: (**a**) masked LST against SI LST, (**b**) masked LST against RF regression LST, and (**c**) masked LST against SFRFR LST.

The distribution histograms of the residuals between the predicted and masked LST are shown in Figure 8. Generally, the error distributions of the SFRFR and RF regression are similar, and most errors are normally distributed from −5 to 5 K, while most errors range from −10 K to 10 K in SI method. By combining Table 3, extreme outliers (with the highest value of −51.01 K and lowest value of 47.18 K) are predicted by the SI model, while the error scales of the other two models remain relatively small. The extreme errors of SFRFR are −7.96 K and 11.1 K, displaying an improvement as the minimum error decreases by 1.85 K and the maximum error decreases by 2.4 K compared with the RF regression results.

**Figure 9:**
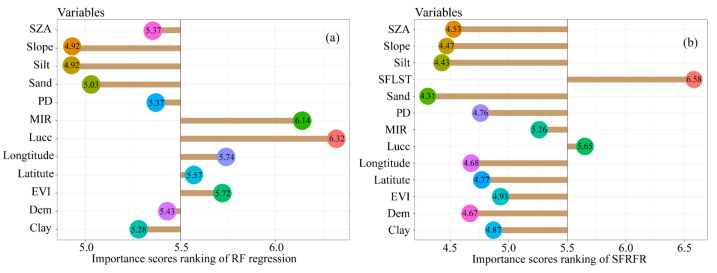
Importance score rankings of the model results: (**a**) RF regression and (**b**) SFRFR.

Additionally, the importance of the explanatory variables obtained for the SFRFR and RF regression results is shown in Figure 9. The importance score rankings indicate the relative contribution of the parameters in the modeling process. To better visualize the ranking of these importance scores, the data are transformed by the common logarithm. In the SFRFR model, $LST_{link}$, LUCC, and MIR are more dominant than the other spatial parameters, with importance scores reaching 6.58, 5.65, and 5.26, respectively. This trend can also be found in the RF regression model, in which the LUCC and MIR had importance scores reaching 6.32 and 6.14, respectively. This shows that the LUCC and MIR are of great importance in modeling. The latitude, longitude, and EVI rank in the second gradient, while the ST (sand, clay, and silt contents) occupies last place.

### 3.4. Performance of Reconstructed Gap-Free LST from 2016 to 2021

**Figure 10:**
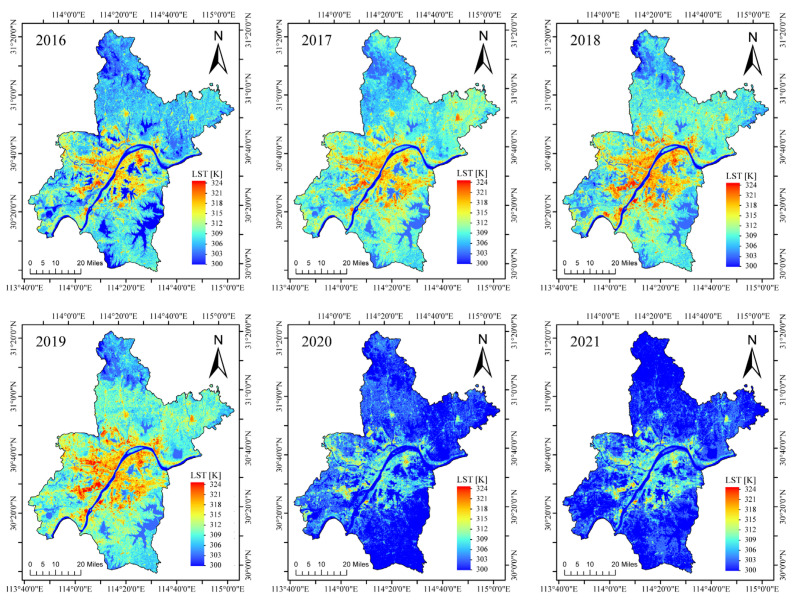
Spatial distribution of the reconstructed summertime LST from 2016 to 2021.

The original images representing the summertime LST from 2016 to 2021 were reconstructed using the proposed model. Figure 10 presents the variations in the urban thermal environment over the past six years. Generally, the thermal emissions showed a decreasing trend from the city centers to the suburbs, and the highest temperature occurred in industrial areas, consistent with a previous study [55]. From 2016 to 2019, urban thermal emissions gradually increased, reflecting in the temperatures in the city center showing rising trends and in the warming of the suburbs. However, in 2020 and 2021, a strong cooling phenomenon occurred. The variations in urban thermal emissions in Wuhan revealed significant temporal and spatial differences.

**Figure 11:**
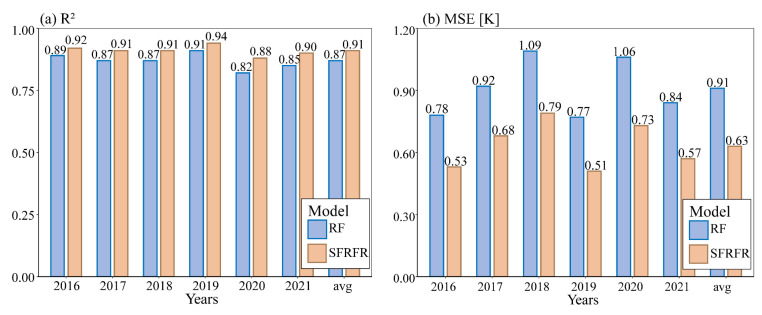
OOB scores of the RF regression and SFRFR models: (**a**) R^2^ values of these two models and (**b**) MSE values of these two models.

Figure 11 illustrates the OOB scores obtained for the two models. The two models have satisfactory results. Compared with the RF regression model, the proposed model presents a better performance in both the R^2^ and MSE scores for each year. On average, the R^2^ value increases by 4%, and the MSE value increases by 28%. Although the enhancement on the R^2^ value is inconspicuous, the intuitive improvement appears on the MSE value. The results indicate that the good results of the proposed model do not represent random events.

**Figure 12:**
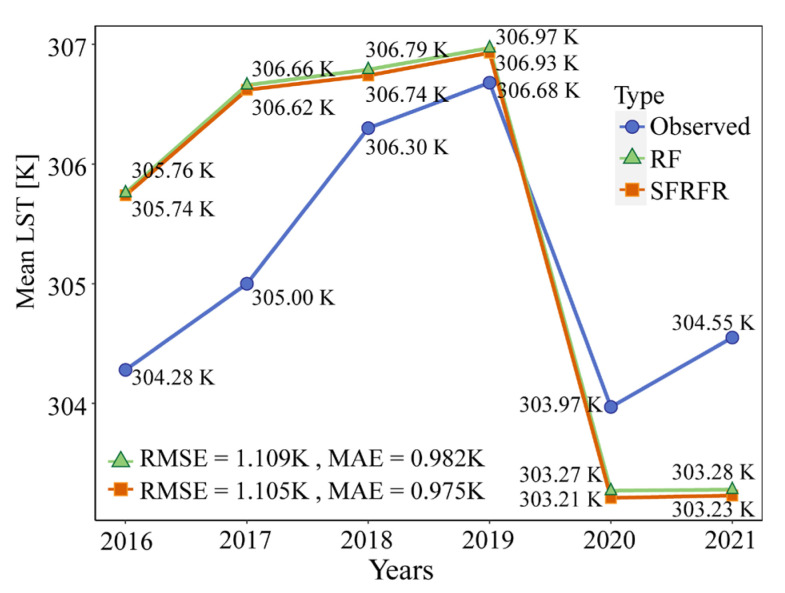
Line chart of the average summertime reconstructed LST and the ground-station-observed LST in the past six years.

Figure 12 shows the variation in the average summertime LST in the predicted data provided by the two models and the ground-station observed data. The SFRFR and RF regression models can simulate the various average LST trends relatively well. From 2016 to 2019, upward trends are presented in the three lines, though the values sharply dropped in 2020. In the last year of study, the trends display rebound effects. In the proposed model, the RMSE and MAE values reached 1.105 K and 0.975 K, respectively, representing improvements of 0.4% and 0.7%, respectively, compared with the RF regression values. Overall, consistent change trends are presented in the predicted and observed LST, exhibiting an increased warming from 2016 to 2019 and cooling phenomena in 2020 and 2021. This trend is also presented in Figure 10, implying that the reconstructed LST data have a decent performance in both their visual effect and accuracy.

### 3.5. The Results of Reconstructed Landsat-LST at 250 m

**Figure 13:**
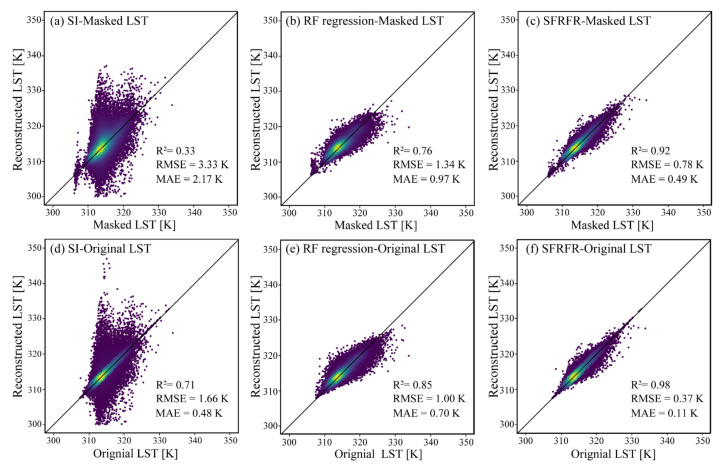
Scatter density plots between the predicted LST and the observed LST under a 250 m spatial resolution: (**a**–**c**) LST plots constructed by the SI, RF regression, and SFRFR models against masked LST regions; (**d**–**f**) LST plots reconstructed by the SI, RF regression, and SFRFR models against original LST regions.

The scatter density plots of predicted LST and observed LST under a 250 m resolution are shown in Figure 13. Overall, the two machine learning-based methods achieve good results and are opposite to the SI method results. With more scatters concentrating on the 1:1 line when compared with the results of RF regression, the SFRFR model achieves the best evaluation metrics. In the masked LST region, the proposed method achieves the highest values of R^2^ (0.92) and lowest values of RMSE (0.78) and MAE (0.49), followed by the RF regression results (R^2^, RMSE, and MAE values of 0.76, 1.34, and 0.97, respectively) and SI method results (R^2^, RMSE, and MAE values of 0.33, 3.33, and 2.17, respectively). Similar to the results at a 100 m spatial resolution, the evaluation metrics of all three methods improve to various degrees in the original LST regions. The SI method gains the most significant improvement, but it still predicts more extreme values than the others. Overall, due to the R^2^ values of 0.98, RMSE values of 0.37, and MAE values of 0.11 in the original LST regions, the SFRFR displays the best results in evaluation metrics.

**Figure 14:**
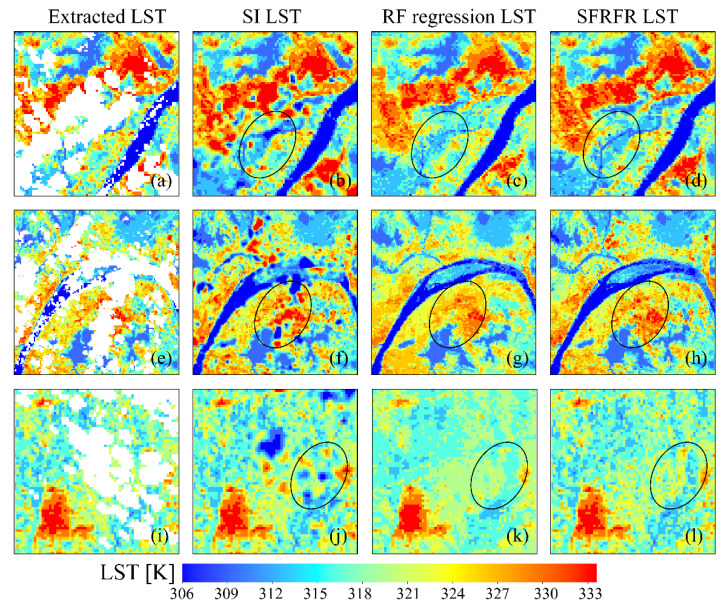
Visual performances of the reconstructed LST and the extracted LST at a 250 m spatial resolution: (**a**) extracted LST, (**b**) SI LST, (**c**) RF regression LST, (**d**) SFRFR LST, (**e**) extracted LST, (**f**) SI LST, (**g**) RF regression LST, (**h**) SFRFR LST, (**i**) extracted LST, (**j**) SI LST, (**k**) RF regression LST, and (**l**) SFRFR LST.

The visual performances of the three predicted LSTs are shown in Figure 14. We randomly choose three subregions in the extracted LST region, including industrial areas with large quantities of thermal emissions (Figure 14a), urban areas with high heterogeneity (Figure 14e), and rural areas with more natural coverages (Figure 14i), to compare the ability of the three methods in simulating the LST. It was found that the SFRFR model presents the most abundant spatial details, whether in urban areas with high heterogeneity or rural areas dominated by natural coverages. In Figure 14d,h,l, the simulated LST presents a spatially steady and distinct variation. The difference between high temperature (red pixels) and sub-high temperature (orange-red pixels) is obvious. The RF regression model still achieves the second-best results. Though the RF regression can fill the gap without eruption, the visual performance performs weaker in some areas. As shown in Figure 14c,g,h in comparison to the SFRFR results, the RF regression possesses fewer and more blurry spatial textures. The transition between high values and sub-high values is unobvious. The SI results are tertiary to the others. It can completely restore the spatial texture of the original LST; however, the method also tends to estimate extreme values when the data are missing to a large extent. This is reflected in the visual performance of the unsteady fluctuation of the LST variation. At the 250 m spatial resolution, the improvement in SFRFR is significant compared with that at the 100 m spatial resolution.

## 4. Discussion

### 4.1. Advantages of the Proposed Model

In this paper, we proposed an algorithm that improved upon RF regression to reconstruct gap-free LST data under a 100 m spatial resolution. Compared with the SI and RF regression results, the SFRFR model produces the best evaluation metrics and visual performance (as shown in Figure 4, Figure 5, and Figure 8). The enhanced performance of SFRFR is mainly a consequence of its ability to build more stable links connecting the LST with the parameters and its incorporation of Kriging regression to reduce residuals. Specifically, the advantages of the proposed method can be divided into the following aspects.

First, the SFRFR model has an improved generalization ability. As the masked LST data make no contribution to the modeling process, the model learns no information from these values. The masked LST instead existed as an independent validation dataset. Combined with Figure 4 and Table 3, when using masked LST to evaluate the model, it can be intuitively seen that the SFRFR model obtains the best evaluation metrics and lowest error range. In terms of its visual performance, as shown in Figure 5, Figure 6 and Figure 7, which possess abundant spatial details, the proposed method also works well in urban areas that are highly heterogeneous. The spatial contribution of LST in urban areas stably varies and presents clear differences from that of surrounding areas with low heterogeneity. The traditional spatiotemporal data fusion method developed by Weng [28] provides good evaluation metrics and visual performance, but Gao’s method performs weaker in areas with high heterogeneity, whereas the SFRFR approach overcomes this limitation.

Second, the SFRFR model establishes more precise links between LST and the related spatial parameters. The LST is an essential surface parameter, and its formation is inseparable from multiple spatial factors, such as the LUCC, ST, and solar radiation. Traditional linear function-based methods, including SI, have weak performances in characterizing spatial LST fluctuations when the study area possesses high spatial heterogeneity; thus, adopting nonlinear relationships between the LST and the related elements to simulate LST variations increases the accuracy of the results. This phenomenon is consistent with the findings of previous studies, which showed that the nonlinear relationships between LST and related parameters are more stable than linear relationships [54]. The unique adaptive splitting mechanism of DTs enables the RF model to better construct nonlinear linkages between the LST and the corresponding spatial parameters. Although both the SFRFR and RF regression models use nonlinear relationships to simulate the LST, the better performance of the SFRFR model may be due to its ability to incorporate the spatial feature of the LST in the model, leading to more spatial information being considered in the model.

Third, the stability and practicability of the SFRFR model are strong. The model was used to reconstruct the summertime LST at a 100 m spatial resolution from 2016 to 2021. Figure 11 and Figure 12 show that the method still has a satisfactory visual performance and evaluation metrics even when the missing ratio exceeds 60%. Overall, the reconstructed LST data retain fine spatial textures and stable spatial variations and can precisely reflect the spatiotemporal distribution of the time series LST. The average OOB scores in the R^2^ results reach 0.91, and diverse repeated experiments guarantee the stability of the SFRFR model. This may be due to abundant multidimensional auxiliary parameters providing more information for reference when the SFRFR model simulates the LST, thus consolidating the spatial links built by the model.

**Figure 15:**
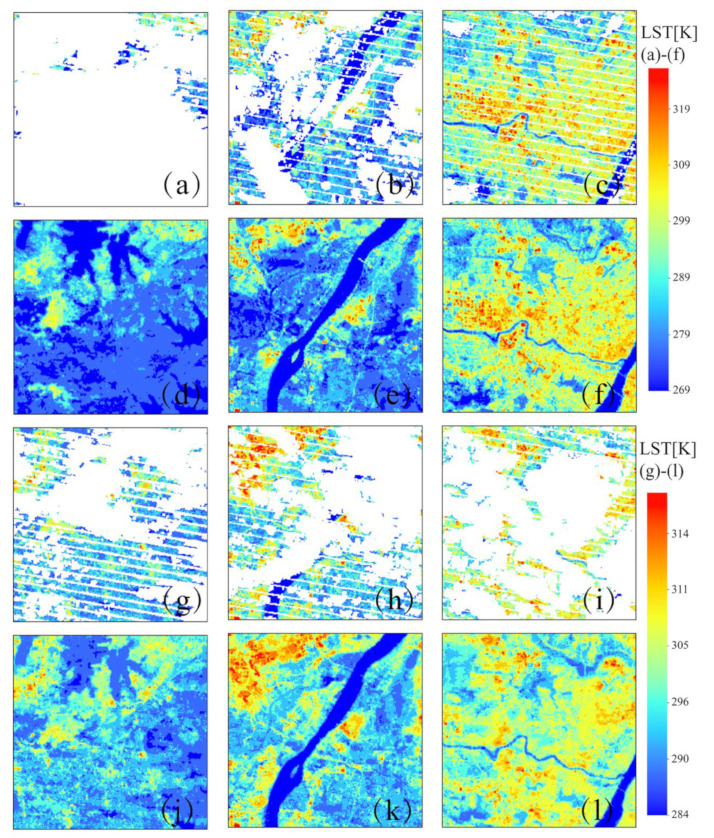
Visual performances of the SFRFR model on 16 June 2017 and 11 August 2020. (**a**–**c**) Regions with different original LST data on 16 June 2017. (**d**–**f**) SFRFR predictions for the corresponding regions on 16 June 2017. (**g**–**i**) Regions that retained different original LST on 11 August 2020. (**j**–**l**) SFRFR predictions of the corresponding regions on 11 August 2020.

Moreover, the SFRFR model makes full use of various information sources derived from multiple sensors with high spatial resolutions to reconstruct the gap-free LST. Specifically, the original LST and auxiliary data are mutually complementary. When the original LST series returns null data over large areas, multidimensional auxiliary data provide rich information as a supplement to the original LST data, allowing the model to simulate the LST. However, some parameters such as the terrain and coordinates are constant and make great contributions to the model. The original LST in turn are used as an anchor to calibrate the fluctuation range of the predictions and avoid extremes. The reconstructions obtained on 16 June 2017 and on 11 August 2020, in which the missing data ratio exceeded 60%, are shown in Figure 15. Figure 15c,i show the original images of the same area on different dates. The first panel shows more of the original LST than the latter. Turning to the predicted results shown in Figure 15f,l, although the missing data in both panels are completely filled with stable LST variations, it is obvious that Figure 15f has more spatial texture and that the spatial distribution of the simulated LST is more consistent with the adjacent original LST, while the result shown in Figure 15i perform weaker. This phenomenon may be due to the diverse range of LST resulting in different symbology classifications generated in ArcGIS, as shown in Figure 15a–c, with the original LST being incrementally retained; the corresponding predictions shown in Figure 15d–f maintain the rich spatial details. It can be inferred that the usage of multidimensional auxiliary data is beneficial for simulating the LST.

### 4.2. Contributions of Parameters Accounted for in the Model

The importance score ranking explains the extent of the contribution of each parameter in the model. As illustrated in Figure 9 above, the importance score of $LST_{link}$ occupied the first ranking in the SFRFR results. Theoretically, spatial continuity and autocorrelation are used for the ideological basis of $LST_{link}$. Each spatial information pixel that strongly correlates with the original LST is constructed by the original adjacent LST pixels. Hence, $LST_{link}$ plays an essential role in the modeling process, which may explain why the nonlinear relationship built by the SFRFR model has superior performance to the other methods in the simulating LST. Different land use cover types represent various underlying surface attributes [45]. Generally, higher thermal phenomena are observed in construction land use types due to their abundant human activities, while cooler thermal phenomena are presented in natural land use types such as grasslands and water due to their transpiration and lack of human interference. The study area contains complicated landscapes and multiple land use cover types. Each different land use cover type reflects the impacts of various underlying factors on the LST. However, because the LUCC data are discrete with eight types, the nonlinear relationship between the LST and coverages is consistent in one unique type, which can be regarded as building a rough frame; other parameters were added to supplement detailed information to make the model more stable and perform better. This may imply the high importance scores of the LUCC in both model results. Although the DEM greatly influenced the modeling process, as has been reported in previous studies [56], the importance of the LUCC represents a more significant impact than that of the DEM in the models assessed in this study. This result may be due to the flat terrain and abundant plains in the study area. It is worth mentioning that the importance score rankings are not the same as the correlation score rankings between the LST and the explanatory variables. The LST is a surface parameter and changes daily; the parameters with finer time resolutions including the SZA, EVI, and MIR band changed as well. In general, the abundant vegetation in summer means a higher value of EVI, which leads to a significant effect in the LST. The EVI may achieve a higher importance score ranking in the model due to its greater contribution and present the opposite results on winter days. In the fields of parameters with low time resolution, such as the LUCC, PD, ST, and DEM, their ability to influence the LST is relatively constant. The contribution of these parameters in the model may be changed due to the variation in the LST and other parameters. For example, the importance score ranking of the LUCC falls to the second when the spatial feature of LST is introduced in the SFRFR model, which means that the importance score rankings of each parameter will change with variations in different parameters and seasons.

### 4.3. Variation in Urban Thermal Emissions from 2016 to 2021 in Wuhan

Generally, from 2016 to 2021, the LST varied, and these variations were accompanied by a trend in which the LST first increased before declining. Consistently, the highest thermal emissions were observed in urban areas, especially industrial regions, while surrounding natural coverage areas exhibited stronger cooling phenomena. The SFRFR model precisely captured this spatial pattern of variations and smoothly displayed the variations by simulating fine-scale LST data, thus maintaining more spatial textures with smaller residuals.

From Figure 12, it can be seen that although the proposed method successfully simulated the time series line chart of the average LST change trend, some residuals still existed. This might be because the original images were polluted by meteorological factors such as clouds and snow, thus resulting in the extreme values presented in the LST retrievals. However, the cloud mask failed to completely remove these erroneous extremes, leading the proposed model to incorrectly learn observations when building the nonlinear links and thus further predict erroneous extremes in the gaps.

### 4.4. The Improvement Gained by the Spatial Feature of the LST

By comparing the reconstructed results at the two spatial resolutions, it is clear that a significant improvement in the predicted performance occurred after introducing the LST spatial feature to the model at the 250 m resolution. This may be due to the reduction in the training sample, which is a common measure to reduce the model complexity and improve its generalization ability. We can infer that good reconstructed results are available solely using the RF model when there is a large dataset. A large quantity of data involved in the model offers abundant information to the model. In this situation, relatively good predicted performances are obtained by only modifying the algorithm’s hyperparameters, including the number of DTs in the forest, training datasets, and features to consider when looking for the best split. Additionally, the process for obtaining the gap-free LST is simplified. Though the SFRFR model can still display better results than RF regression, it will spend more time in the training of the model and predicting the LST with an insignificant improvement compared with the latter model. In other cases, introducing LST spatial features is effective at improving the model performance. We determine that this may be because the limited sample dataset constrains the model to learn more essential spatial messages. The original resolution of the LUCC and DEM are finer than *SFLST*; however, there exists a data loss when they are aggregated to another spatial resolution. Compared with the LUCC and DEM, *SFLST* provides correct and complete information because it has the same spatial resolution as the original LST. This allows the model to learn more precise spatial messages. The inclusion of *SFLST* data in the model is valuable because of its strong correlation with the LST, which allows it to provide valuable information about spatial variations that may impact the predictions. Specifically, the LST is a useful metric for understanding the spatial distribution of heat in an area, with higher values typically observed in urban industrial areas and lower values observed in natural areas such as parks and green spaces. This variation in the LST is reflected in the spatial characteristics of *SFLST*, which takes into account the spatiotemporal consistency and autocorrelation of the LST. Unlike other auxiliary data, *SFLST* provides detailed information about the spatial textures of LST, allowing the model to better capture the nuances of the data and improve its ability to generalize and accurately estimate missing values. However, in some areas of *SFLST*, the transitions between different values may not be clear, which can result in less accurate predictions. By incorporating additional auxiliary data such as the LUCC and DEM, the model can better capture the nuances of spatial textures and improve its overall accuracy and reliability. This enhances the predictive power of the model. Hence, the introduction of *SFLST* is beneficial for improving the performance of the model, especially when the samples are limited.

### 4.5. Limitations

Although the proposed method shows satisfactory results in restoring the gap-free LST, some limitations still exist. Overall, the visual performance of the predictions built by the SFRFR model may be limited by the quality of the input data, especially the original LST. When missing data in the original LST series are clustered and continuous in a given area, even though the abundant auxiliary parameters are used as reference information for the model, the visual performance of the reconstructed LST in these areas still needs to be improved due to the insufficient original data. From the importance scores ranking of the SFRFR and RF regression model, the spatial features of the LST, LUCC, and MIR all make great contributions to the modeling process. However, the spatial resolution of the MIR reaches only 250 m, which is coarser than the original 100 m resolution. The LUCC data have a 30 m spatial resolution, but these data are discrete with only seven classifications, implying that their ability to characterize the LST are weaker in highly heterogeneous areas. The Kriging method is used to estimate the residuals, but it works weakly in areas with fewer effective messages. In future work, we will focus on identifying an appropriate method for predicting residuals with fewer extremes. The *SFLST* is calculated by building a stable, nonlinear relationship between the LST and the parameters, including the MIR and LUCC, through RF regression, which means that the LST spatial feature is also affected by the quality of the MIR and LUCC data. Considering the selected parameters with high spatiotemporal resolutions may be helpful for refining the predicted visual results.

## 5. Conclusions

Influences such as sensor limitations and meteorologic factors often restrain the generation of continuous high spatiotemporal resolution land surface parameters such as the LST. In this study, by improving upon the RF regression method, we propose the SFRFR algorithm to simulate the spatial distribution and detailed texture of the original LST by constructing a stationary nonlinear relationship between the LST and related variables. The gap-free LST were reconstructed in July 2017 in Wuhan. Comparing the results to those obtained with the RF regression and SI methods, the proposed method was found to possess the best generalization ability. The evaluation metrics of the proposed method include an R^2^ value of 0.96, an RMSE value of 0.55, and an MAE value of 0.18, thus attaining the best effect among the three models. With regards to its visual performance, the results show that the SFRFR model precisely captures thermal variations and maintains the most spatial details among the assessed models.

By restoring the time series summertime LST data from 2016 to 2021 in Wuhan, we found that the SFRFR also has good stability and applicability. In diverse repeated modeling, the average R^2^ value remained at 0.91, and the MSE remained at 0.63. Compared with the LST recorded at ground stations, the RMSE was only 1.105 K, and the MAE was 0.975 K; these are acceptable errors. Nevertheless, we also found that the visual prediction effect was dominated by the amount of original LST data input and the quality of the auxiliary data. Weaker performances often appeared in areas with little original information and worse-quality auxiliary parameters.

Overall, the SFRFR model is a stable and practical algorithm for acquiring gap-free data. In the nonlinear relationships built by this model, the dependent variables are not limited to the LST. Other surface parameters, such as soil moisture and precipitation, can also be applied. This method can be widely used in time series urban heat island monitoring, fine-scale surface parameter reconstructions, and other fields.

## Figures and Tables

**Table 1:** The sources of LST data and the related spatial parameters.

Data	Sources	Spatial Resolution (m)	Time Resolution (d)	Acquisition Date
Practical single-channel application land surface temperature (PSC APP LST)	Landsat 8	100	16	26 July 2017
Enhanced vegetation index (EVI)	MOD13Q1	250	16	27 July 2017
Solar zenith angle (SZA)	MOD13Q1	250	16	27 July 2017
Middle-infrared surface reflectance (MIR)	MOD13Q1	250	16	27 July 2017
Land use and land cover change (LUCC)	Zenodo	30	365	2017
Population density (PD)	Worldpop	100	365	2017
Soil texture (ST)	SoilGrids250m	250	/	2017
Digital elevation model (DEM)	ALOS	12.5	/	2011
Slope	ALOS	12.5	/	2011

**Table 2:** Characteristics of LST data remaining to be reconstructed.

Acquisition Date	Source	Sensor	Data Loss (%)
05 Jun 2016	Landsat 8	TIRs	13.72
23 July 2016	Landsat 8	TIRs	2.06
01 Sep 2016	Landsat 7	ETM+	21.07
16 Jun 2017	Landsat 7	ETM+	66.72
18 July 2017	Landsat 7	ETM+	39.20
27 Aug 2017	Landsat 8	TIRs	4.80
03 Jun 2018	Landsat 7	ETM+	53.09
21 July 2018	Landsat 7	ETM+	23.13
29 July 2018	Landsat 8	TIRs	10.00
14 Jun 2019	Landsat 8	TIRs	26.08
30 Jun 2019	Landsat 8	TIRs	13.19
17 Aug 2019	Landsat 8	TIRs	3.00
08 Jun 2020	Landsat 7	ETM+	28.15
11 Aug 2020	Landsat 7	ETM+	63.06
27 Aug 2020	Landsat 7	ETM+	29.38
29 July 2021	Landsat 7	ETM+	30.51
06 Aug 2021	Landsat 8	TIRs	39.52
30 Aug 2021	Landsat 7	ETM+	30.70

**Table 3:** Errors between the results of the three models and the original LST.

Model	Differences in Quantitative Error Indicators (K)					
	Min	1-st Qu	Med	Mean	3-rd Qu	Max
SFRFR	−7.96	−0.70	−0.10	−0.05	0.52	11.1
RF regression	−9.81	−0.76	−0.13	−0.05	0.55	13.5
SI	−51.01	−1.11	−0.02	−0.02	1.07	47.18

## Data Availability

All the datasets used in this study are freely available.
